# Online Patient Education Materials for Common Sports Injuries Are Written at Too-High of a Reading Level: A Systematic Review

**DOI:** 10.1016/j.asmr.2021.12.017

**Published:** 2022-02-11

**Authors:** Youssef Abdullah, Aaron Alokozai, Samantha O'Connell, Mary K. Mulcahey

**Affiliations:** a Department of Orthopaedic Surgery (M.K.M.), Tulane University School of Medicine, New Orleans, Louisiana, USA; b Office of Academic Affairs and Provost, Tulane University, New Orleans, Louisiana, USA

## Abstract

**Purpose:**

To determine the readability of online patient information for common sports injuries.

**Methods:**

A systematic search of the literature using PubMed/MEDLINE, Embase, and the CINAHL databases was performed according to Preferred Reporting Items for Systematic Reviews and Meta-Analysis guidelines. Studies were included if they (1) were published between 2000 and September 2020, (2) were English-language publications and complete studies from peer-reviewed journals, (3) evaluated online information directed toward patients with common sports injuries.

**Results:**

Eleven studies met inclusion criteria and were included. The mean Flesch-Kincaid Grade Level for online education information was 10.5, whereas the mean Flesch Reading Ease was 51.2, indicating existing health resources are written above the recommended readability grade level (no greater than a sixth-grade reading level). The mean DISCERN score was 41.5, indicating that the quality of information accessible to patients was fair. The accuracy of health content determined by the ACL-Specific Score was reported as moderate level (mean 8.85).

**Conclusions:**

This study demonstrates that online patient information regarding common sports injuries the does not match the readability recommendations of the American Medical Association and National Institutes of health.

**Clinical Relevance:**

Future health-related information should be written by qualified experts at a level that can be easily understood by patients of all health literacy levels. Surgeons should be more attentive to where patients get their information from and how they interpret it. Accurate, easy to understand educational tools can improve efforts to help patients identify misconceptions about treatment options, and to guide patients to choices that are consistent with their values.

The value of patient education materials relies on the users’ ability to access and understand the presented information. Within the last several years, the Internet has transformed into the primary source of health information for many people.^1^ More than 345 million Americans, representing 95.0% of the population, have Internet access, with more than one-half using the Internet to seek health information.^2^

Moreover, there is an emergent body of literature across multiple specialties supporting the importance of accurate and accessible health information for patients.^3^ The quality of information provided to patients regarding their care may substantially influence their understanding of their condition/injury.^4^ Further, patient education may influence treatment choice and outcome expectations.^5^ In the orthopaedic setting, effective patient education may contribute to a favorable postoperative course. Johansson et al.^6^ reported that preoperative orthopaedic patient education improved pain, length of hospital stay, self-efficacy, and motivation to complete exercises. It is therefore imperative to assess the quality, readability, and accuracy of online patient education materials. Furthermore, patient education tools are now a major focus in management and are counted among the factors considered in health care quality assessment.^7^ Attention to from where patients obtain their information and how they interpret it represents an important step in patient management: the patient, when correctly informed, plays a substantial role in discussing treatment options and subsequent surgical procedures.^8^^,^^9^ Without quality information, the patient is in less of a position to accurately weigh tests and treatment options that are in line with their goals, values, and preferences.^9^ The purpose of this study was to determine the readability of online patient information for common sports injuries. We hypothesized that the readability of online patient information for common sports injuries would not meet recommended levels.

## Methods

The systematic review was performed in accordance with the Preferred Reporting Items for Systematic Reviews and Meta-Analysis (PRISMA) guidelines.^10^ No meta-analysis was undertaken for the included studies, given the heterogeneity of patient education materials assessed.

### Information Sources and Search Strategy

The literature search was conducted, with the assistance of a research support librarian, using the PICO framework. A comprehensive search was conducted using the PubMed/MEDLINE, Embase, and CINAHL databases. All databases were searched from inception to September 2020. Each database was searched for the following Medical Subject Headings (MeSH) and key words: “athletic injuries,” “education delivery,” “patient engagement,” “shared decision-making,” “preoperative,” and “postoperative.” Search and query of terms used in combination with Boolean operators available as Appendix Tables 1-3, available at www.arthroscopyjournal.org. Each included study’s reference list was also reviewed.

### Eligibility Criteria

Studies were included if they (1) were published between September 2000 and September 2020 to capture different variations of studies, while excluding obsolete knowledge, and incorporating the present trends in the study topic as compared to the recent past; (2) were English-language publications and complete studies from peer-reviewed journals; and (3) evaluated online information directed toward patients with common sports injuries. Exclusion criteria were publication types other than peer-reviewed studies such as protocols, reviews, or case series.

### Selection Process and Data Collection

The query yielded 722 studies from PubMed/MEDLINE, 2868 from Embase, and 3652 from CINAHL databases after duplicates were removed. Data were independently extracted by 2 of the coauthors (Y.A. and A.A.) using standard data extraction forms for all studies. These reviewers screened full-text studies using the same procedure with acceptable reproducibility for all decisions. Disagreements were resolved by consensus. The following data items were collected: condition or injury, information source, number of webpages analyzed, authorship, methods of acquiring information, and key study results regarding quality, readability, and accuracy (Table 1).^11^, ^12^, ^13^, ^14^, ^15^, ^16^, ^17^, ^18^, ^19^, ^20^, ^21^

### Outcome Measures

#### Measures of Readability

Three scores were used to calculate readability: Flesch-Kincaid Grade Level (FKGL), Flesch Reading Ease Score (FRES), and Gunning Fog Index (GFI). FKGL measures the grade level that one must complete to comprehend a given text, whereas the FRES measures the readability of a text.^22^ FKGL and FRES range from 0 to 29: very difficult to read or a postgraduate reading level; 30 to 49: difficult to read, college reading level; 50 to 59: fairly difficult to read, high school reading level; 60 to 69: standard difficulty to read, 8th to 9th grade reading level; 70 to 79: fairly easy to read, 7th grade reading level; 80 to 89: easy to read, 5th to 6th grade reading level; 90 to 100: very easy to read, 4th to 5th grade reading level. GFI estimates the years of formal education a person needs to understand the text on first reading.^23^, ^24^, ^25^

### Measures of Quality and Accuracy

Six scores were used to calculate quality and accuracy: DISCERN questionnaire, *Journal of the American Medical Association* (*JAMA*) benchmark criteria, ACL Specific Score (ASS), the Global Quality score (GQS), Unique quality and accuracy score, and Health On the Net Code (HONcode).

The DISCERN questionnaire is a standardized quality index of consumer health information that determines publication quality based on 16 questions that pertain to the reliability of the publication, content information, and overall quality rating.^26^ The DISCERN criteria scale ranges from 6-80, with a greater score indicating greater quality.

The *JAMA* benchmark criteria assesses 4 core criteria to determine whether the information presented was credible, reasonable, or potentially useable.^12^ The *JAMA* benchmark criteria scale ranges from 0 to 4, with a greater score indicating greater quality.^12^

The ASS, defined by Bruce-Band et al.,^12^ evaluates informational value of each website pertaining to ACL injuries and reconstruction. One point was assigned for each criterion, with a potential score of 25. The ASS is scored as very good (21-25), good (16-20), moderate (11-15), poor (6-10), or very poor (0-5).

The GQS was assigned by the reviewer after evaluating the pertinent websites. The GQS uses a 5-point scale to rate overall quality and scores range from 0 to 5, with a greater score indicating greater quality.

Unique quality and accuracy scores are based on guidelines written by American Academy of Orthopedic Surgeons (greater score = greater quality or accuracy).^12^, ^13^, ^14^^,^^16^^,^^17^^,^^27^^,^^28^

Finally, the presence of HONcode certification identifies websites that agree to comply with a code of ethics to provide quality objective and transparent medical information.^29^

### Assessment of Study Quality

Study quality was evaluated through the following variables recommended in Crombie’s items for assessing the quality of cross-sectional studies^30^: (1) appropriateness of design to meet the aims, (2) justification of sample size, (3) adequate description of the data, (4) report number of excluded studies, (5) adequate representativeness of the sample to the total, (6) clearly stated aims and likelihood of reliable and valid measurements, and (7) adequate description of statistical methods. Each parameter received a score of 0, 0.5, or 1 point for not reporting, unclearly reporting, or clearly reporting, respectively. Studies were denoted as high quality if more than 5 of the 7 criteria were described and considered. Studies were denoted as moderate quality if 4-5 of the criteria were described and considered. Quality scores less than 4 were deemed low quality.

## Results

**Fig 1:**
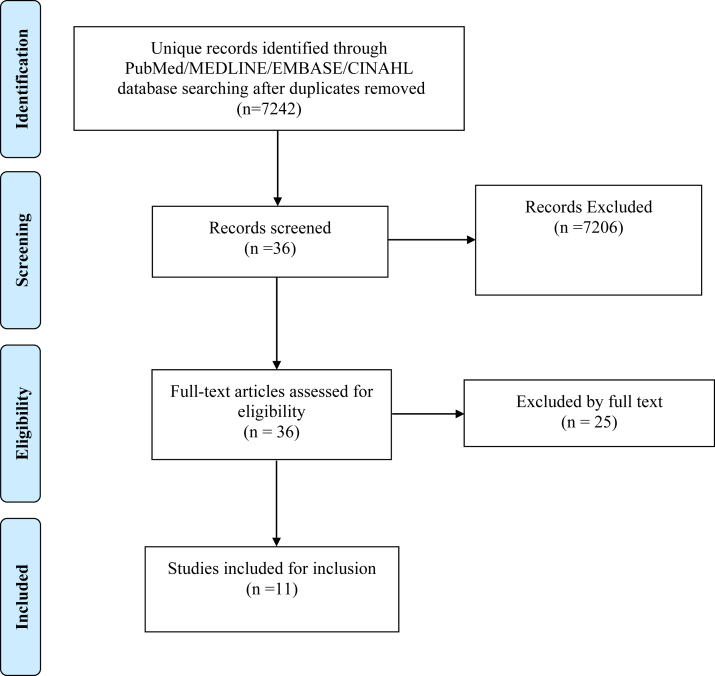
Preferred Reporting items for Systematic Review and Meta-Analysis Statement Diagram depicting the selection process for article inclusion.

The query yielded 722 studies from PubMed/MEDLINE, 2868 from Embase, and 3652 from CINAHL databases after duplicates were removed. Applying inclusion and exclusion criteria resulted in 11 studies included for analysis (Fig 1). The article characteristics are included as a tabulated and narrative summary (Table 1).^11^, ^12^, ^13^, ^14^, ^15^, ^16^, ^17^, ^18^, ^19^, ^20^, ^21^ The most common sports injury studied was ACL tear.^7^^,^^11^, ^12^, ^13^^,^^15^^,^^21^ Other injuries included meniscus tear,^9^^,^^20^ hip labral tear,^11^ shoulder labral tear,^11^ rotator cuff,^14^^,^^18^^,^^31^^,^^32^ ulnar collateral ligament tear,^17^ articular cartilage defects,^33^ shoulder instability,^16^ and ankle fractures.^34^ Eight studies assess education websites, 2 assess YouTube videos, and 1 assess web-based protocols. Physician authorship ranged from 2% to 39%. The number of websites/videos/protocols evaluated in each study ranged from 30 to 200.

**Table 1:** Characteristics of Studies Included in the Systematic Review

Study	Study Design, Level of Evidence	Information Source(s)	Outcome(s)	Condition or Injury	Conclusion
Akinleye et al., 2018^11^	Retrospective, IV	Educational websites	Readability scores	ACL tear, meniscus tear, hip labral tear, rotator cuff tear	Most frequently accessed materials for patients with injuries requiring arthroscopic surgery does not match the readability recommendations of the AMA and NIH, and the average reading ability of U.S. adults.
Bruce-Brand et al., 2013^12^	Retrospective, IV	Educational websites	Quality scores	ACL reconstruction	Quality of information available online regarding ACL reconstruction is of variable quality with many websites omitting basic information regarding treatment options, risks, and prognosis.
Cassidy et al., 2018^13^	Retrospective, IV	YouTube videos	Quality scores	ACL injury and reconstruction	Majority of videos viewed on YouTube regarding ACL injury and treatment are of low quality
Dalton et al., 2015^14^	Retrospective, IV	Educational websites	Readability and quality scores	Rotator cuff tears	Quality of available information on rotator cuff tears is poor. Readability of information on rotator cuff disease is inappropriately high.
Duncan et al., 2013^15^	Retrospective, IV	Educational websites	Proportion of websites that met prespecified quality criteria	ACL reconstruction	Quality of internet information available to patients searching for ACL reconstruction appears mixed.
Garcia et al., 2014^16^	Retrospective, IV	Educational websites	Readability, quality, and accuracy scores	Shoulder instability	Online information regarding shoulder instability is often inaccurate and/or at an inappropriately high reading level. The quality of information is highly dependent on the specific search term used.
Johnson et al., 2016^17^	Retrospective, IV	Educational websites	Readability, quality, and accuracy scores	Ulnar collateral ligament	Online information on UCL injuries is often inaccurate and written at an inappropriate reading level. Information quality depends on search term used, website authorship, and commercial bias.
Lawson et al., 2016^18^	Retrospective, IV	Educational websites	Readability and quality scores	Rotator cuff repair	Websites associated with academic institutions produced the highest-quality medical information.
Wang et al., 2017^19^	Retrospective, IV	Educational websites	Readability, quality, and accuracy scores	Articular cartilage defects	Quality and readability of online patient resources for articular cartilage defects favor those with a higher level of education. Majority of websites do not distinguish between focal chondral defects and diffuse osteoarthritis, which can fail to provide appropriate patient education and guidance for available treatment.
Trofa et al., 2019^20^	Retrospective, IV	Web-based protocols	Proportion of protocols that met prespecified quality criteria	Isolated meniscal repairs	Within the most readily available online protocols there are significant disparities in regard to brace use, ROM, weight-bearing, and strengthening and proprioception exercises.
Springer et al, 2020^21^	Retrospective, IV	YouTube videos	Quality scores	ACL reconstruction	Average information quality, reliability and accuracy of YouTube videos regarding rehabilitation and RTS after ACL reconstruction are poor. Information quality of related YouTube videos from medically trained professionals is significantly higher compared with commercial videos or personal-testimony videos

ACL, anterior cruciate ligament; AMA, American Medical Association; NIH, National Institutes of Health; RTS, return to sport; UCL, ulnar collateral ligament.

### Readability, Quality, and Accuracy of Information

Table 2^11^, ^12^, ^13^, ^14^, ^15^, ^16^, ^17^, ^18^, ^19^, ^20^, ^21^ reports the readability, quality, and accuracy of online patient information for sports medicine related injuries. Six of 11 (54.5%) studies evaluated components of readability (Table 3).^11^^,^^14^^,^^16^, ^17^, ^18^, ^19^ The mean FKGL was 10.5 (range 8.1-13.4), which is defined as “very difficult to read,” or a postgraduate reading level. The mean FRES was 51.18 (range 50.17-52.14), which is defined as “fairly difficult to read,” or a high school reading level. Only one study reported a mean GFI of 9.02, which is higher than the threshold (index less than 8) for universal understanding.^19^

**Table 2:** Characteristics of Online Education Materials

Citation	Condition or Injury	N	Method	Search Engines	Search Terms	% Physician Authored	Outcome Scores, Mean (SD)
Readability							
Akinleye et al., 2018^11^	ACL tear, meniscus tear, hip labral tear, shoulder labral tear, and rotator cuff tear	50	10 most-visited sites for each condition were analyzed.	Google	ACL tear, meniscus tear, rotator cuff tear, shoulder labral tear, and hip labral tear	16% private practice	FKGL, 9.0 FRES, 52.14
**Quality**							
Bruce-Brand et al., 2013^12^	ACL tear, meniscus tear, hip labral tear, shoulder labral tear, and rotator cuff tear	45	Reviewed first 30 results from Google, 10 from Yahoo, Bing, and Ask.	Google, Yahoo, Bing, Ask	ACL reconstruction	11% physician	DISCERN, 41.11 (13.3) *JAMA*, 2.1 (1.2) HONcode-certified (18%) Quality score, 12.29 (5.49); scale 0-25
Cassidy et al., 2018^13^	ACL injury and reconstruction	39	Considered results from only first three pages for each search.	YouTube	ACL, ACL with/without associated terms of injury, reconstruction, and surgery	2% private practice	DISCERN, 2.2 (0.9); modified scale 0-5 *JAMA*, 2.4 (0.7) ASS, 5.5 (3.2)
Duncan et al., 2013^15^	ACL reconstruction	200	Identified top 50 sites from each of the 4 search engines.	Google, Yahoo, Bing, Ask	ACL reconstruction	36% private physician or physician groups with no academic affiliation	(41.5%) had ability to contact author, (60%) had discussion of disorder, (31%) had treatment options, (29%) explained eligibility for ACL reconstruction, (20.5%) mentioned related injuries, (62.5%) reported surgical technique, (55%) mentioned graft selection, (30%) included complications, (48.5%) discussed rehabilitation, (26%) had peer-reviewed references
Trofa et al., 2019^20^	Isolated meniscal repairs	30	Twenty official meniscal repair rehabilitation protocols identified through the Electronic Residency Application Service and first 10 protocols identified by the Google search were included.	Electronic Residency Application Service, Google	Meniscal repair physical therapy protocol	–	(86.6%) recommended immediate postoperative bracing; (40.0%) permitted immediate weight-bearing as tolerated (WBAT) postoperatively, remaining protocols permitted WBAT at an average of 4.0 (range, 1-7) weeks. Most protocols (73.3%) initiating immediate passive ROM to 90°. Only 5 protocols (16.7%) employed functional testing as a marker for return to athletics.
Springer et al., 2020^21^	Anterior cruciate ligament reconstruction	140	Use of Onion Router software for nonbiased search results. Only videos within first 3 pages were included in the analysis. Analyzed information on rehabilitation and return to sport.	YouTube	**Rehabilitation:** ACL rehab, ACL rehabilitation, ACL rehabilitation protocol, ACL rehabilitation program, rehab ACL surgery; **Return to sport:** return to sport after ACL reconstruction, ACL surgery return to sport, return to sport after ACL surgery, return to play after ACL surgery, return to play after ACL reconstruction	Rehabilitation: 13.6% educational physician RTS: 23.2% educational physician	**Rehabilitation:** *JAMA*, 1.32 (0.64) GQS, 1.95 (1.1) Quality score, 5.0 (3.4); scale 0-20 **RTS:** *JAMA*, 1.6 (0.7) GQS, 1.6 (0.8) Quality score, 3.1 (3.4); scale 0-20
Readability and quality							
Dalton et al., 2015^14^	Rotator cuff tears	59	Top 25 results from each search engine were analyzed.	Top 5 search engines	Rotator cuff tear	36% physician/surgeon	FKGL, 8.10 (1.74) FRES, 51.24 (11.42) GFI, 9.02 (2.34) DISCERN, 39.47 (11.39) *JAMA*, 1.72 HONcode-certified (25%)
Lawson et al., 2016^18^	Rotator cuff repair	150	Top 50 sites from each website were identified. Searched at 2 time points: 2011 and 2014.	Google, Yahoo, Bing	Rotator cuff repair	Time 1 (2011): 38% private practice Time 2 (2014): 38% private practice	FKGL, 10.98 FRES, 50.17 DISCERN, 44 HONcode-certified (11%)
Readability, quality, and accuracy							
Garcia et al., 2014^16^	Shoulder instability	82	Evaluated the first 25 results from each search.	Google, Yahoo, Bing	Shoulder instability, loose shoulder, and shoulder dislocation	16% physician with academic affiliation 39% physician without academic affiliation	FKGL, 10.96 (2.5) Quality score, 9.48 (5.11); scale 0-25 Accuracy score, 8.61 (2.6); scale 0-12
Johnson et al., 2016^17^	UCL injuries	113	Evaluated the first 25 results from each search.	Google, Yahoo, Bing	Elbow ulnar collateral ligament injury, tommy john injury, and pitcher's elbow	29% physician	FKGL, 10.71 (2.6) *JAMA*, 1.72 HONcode-certified (3.5%) Quality score, 8.8 (6.8); scale 0-32 Accuracy score, 6.26 (2.9); scale 0-12
Wang et al., 2017^19^	Articular cartilage defects	53	First 25 results from each engine were collected and reviewed.	Google, Yahoo, Bing	Cartilage defect, cartilage damage, cartilage injury	–	FKGL, 13.4 (8.0) Quality score, 7.4 (4.4); scale 0-25 Accuracy score, 11.7 (0.6); scale 0-12

NOTE. FKGL: Flesch-Kincaid Grade Level measures grade level one must complete to comprehend a given text. FRES: Flesch Reading Ease Score measures readability of a text. Score 0-29: very difficult, postgraduate; 30-49: difficult, college; 50-59: fairly difficult, high school; 60-69: standard, 8th to 9th grade; 70-79: fairly easy, 7th grade; 80-89: easy, 5th to 6th grade; 90-100: very easy, 4th to 5th grade. GFI: Gunning Fog Index estimates years of formal education a person needs to understand the text on first reading. DISCERN questionnaire is a standardized quality index of consumer health information. Scale 6-80 (greater score = greater quality). *JAMA* (Journal of the American Medical Association) benchmark criteria. Scale 0-4 (greater score = greater quality). ASS (ACL-Specific Score) scores as very good (21-25), good (16-20), moderate (11-15), poor (6-10), and very poor (0-5). GQS (Global Quality Score) scale 0-4 (greater score = greater quality). Unique quality and accuracy scores based on guidelines written by American Academy of Orthopedic Surgeons (higher score = higher quality or accuracy). Scores vary by condition.

ACL, anterior cruciate ligament; RTS, return to sport; SD, standard deviation; UCL, ulnar collateral ligament.

**Table 3:** Readability Scores

Study	Mean FKGL (SD)	Mean FRES (SD)	Mean GFI (SD)
Akinleye et al., 2018^11^	9.00	52.14	–
Dalton et al., 2015^14^	8.10 (1.74)	51.24 (11.42)	9.02 (2.34)
Garcia et al., 2014^26^	10.96 (2.5)	–	–
Johnson et al., 2016^17^	10.71 (2.6)	–	–
Lawson et al., 2016^18^	10.98	50.17	–
Wang et al., 2017^19^	13.40 (8.0)	–	–
Average	10.52	51.18	9.02

NOTE. FKGL: Flesch-Kincaid Grade Level measures grade level one must complete to comprehend a given text. FRES: Flesch Reading Ease Score measures readability of a text. Score 0-29: very difficult, postgraduate; 30-49: difficult, college; 50-59: fairly difficult, high school; 60-69: standard, 8th to 9th grade; 70-79: fairly easy, 7th grade; 80-89: easy, 5th to 6th grade; 90-100: very easy, 4th to 5th grade. GFI: Gunning Fog Index estimates years of formal education a person needs to understand the text on first reading.

SD, standard deviation.

Ten of 11 (90.9%) studies evaluated components of quality (Table 4).^12^, ^13^, ^14^, ^15^, ^16^, ^17^, ^18^, ^19^, ^20^, ^21^ Overall, the quality of information accessible to patients was classified as fair, with a mean DISCERN score of 41.5 (range 39.47-44). The mean *JAMA* benchmark score for websites was 1.8 (range 1.32-2.4). Only one study reported a poor ASS of 5.5.^18^ Bruce-Band et al.^12^ and Dalton et al.^19^ demonstrated that HONcode-certified sites (2 studies in total), were significantly more difficult to read (*P* = .004).

**Table 4:** Quality Scores

Study	Mean DISCERN (SD)	Mean *JAMA* (SD)	Mean ASS (SD)	Mean GQS (SD)	HONcode-Certified, no., %	Mean Unique Quality Score (SD)
Bruce-Brand et al., 2013^12^	41.10 (13.3)	2.10 (1.2)	–	–	8, 18%	12.29 (5.49) scale 0-25
Cassidy et al., 2018^13^	2.20 (0.9)∗ modified DISCERN scale (0-5)	2.40 (0.7)	5.50 (3.2)	–	–	–
Dalton et al., 2015^14^	39.47 (11.39)	1.72	–	–	15, 25%	–
Duncan et al., 2013^15^						
Garcia et al., 2014^16^	–	–	–	–	–	9.48 (5.11) scale 0-25
Johnson et al., 2016^17^	–	1.43	–	–	4, 3.5%	8.80 (6.8) scale 0-32
Lawson et al., 2016^18^	44.00	–	–	–	12,11%	–
Wang et al., 2017^19^	–	–	–	–	–	7.40 (4.4) scale 0-25
Trofa et al., 2019^20^	–	–	–	–	–	–
Springer et al., 2020^21^	–	Rehabilitation after ACLR: 1.32 (SD, 0.64) RTS after ACLR: 1.6 (SD, 0.7)	–	Rehabilitation after ACLR: 1.95 (SD, 1.1) RTS after ACLR: 1.6 (SD, 0.8)	–	Rehabilitation: 5.00 (SD, 3.40); RTS 3.10 (SD, 3.40) scale 0-20
Average	41.52	1.79	5.50	1.95	9.75	8.60

NOTE. The DISCERN questionnaire is a standardized quality index of consumer health information. Scale 6-80 (greater score = greater quality). JAMA (Journal of the American Medical Association) benchmark criteria. Scale 0-4 (greater score = greater quality). ASS (ACL Specific Score) scores as very good (21-25), good (16-20), moderate (11-15), poor (6-10), and very poor (0-5). GQS (Global Quality Score) scale 0-4 (greater score = greater quality). Asterisk indicates that a modified discern scale was used. Please move the text following the asterisk to the bottom of the table “modified DISCERN scale (0-5)”.

SD, standard deviation.

Three of eleven (27.3%) studies evaluated accuracy (Table 5).^16^^,^^17^^,^^19^ Overall, the accuracy of information was moderate (mean 8.85, range 6.26-11.7).^16^^,^^17^^,^^19^

**Table 5:** Accuracy Scores

Study	Mean Unique Accuracy Score (SD)	Scale
Garcia et al., 2014^16^	8.61 (2.6)	0-12
Johnson et al., 2016^18^	6.26 (2.9)	0-12
Wang et al., 2017^19^	11.70 (0.6)	0-12
Average	8.86	

NOTE. Unique quality and accuracy scores based on guidelines written by American Academy of Orthopaedic Surgeons (greater score = greater quality or accuracy). Scores vary by condition.

SD, standard deviation.

### Assessment of Study Quality

Study quality of articles included in the review ranged from 4.5 to 7, indicating moderate to high quality. Fifteen of 17 studies (88.2%) were high quality based on their quality assessment scores, whereas 2 of 17 (11.8%) were moderate quality. No studies were deemed low quality (Table 6).^11^, ^12^, ^13^, ^14^, ^15^, ^16^, ^17^, ^18^, ^19^, ^20^, ^21^

**Table 6:** Assessment of Study Quality

Study	Appropriateness of Design to Meet the Aims	Justification of Sample Size	Adequate Description of the Data	Report Number of Excluded Results	Adequate Representativeness of the Sample to the Total	Clearly Stated Aims and Likelihood of Reliable and Valid Measurements	Adequate Description of Statistical Methods	Total
Cailliez et al., 2012^7^	Yes	Yes	Yes	Yes	Unclear	Yes	Yes	High
Akinleye et al., 2018^11^	Yes	No	Yes	Yes	Unclear	Yes	Yes	High
Bruce-Brand et al., 2013^12^	Yes	No	Yes	Yes	Yes	Yes	Yes	High
Cassidy et al., 2018^13^	Yes	Yes	Yes	Yes	Yes	Yes	Yes	High
Dalton et al., 2015^14^	Yes	Unclear	Yes	Yes	Unclear	Yes	Yes	High
Duncan et al., 2013^15^	Yes	No	Yes	Yes	Unclear	Unclear	Unclear	Moderate
Garcia et al., 2014^16^	Yes	No	Yes	Yes	Unclear	Yes	Yes	High
Johnson et al., 2016^17^	Yes	No	Yes	Yes	Unclear	Yes	Yes	High
Lawson et al., 2016^18^	Yes	No	Yes	Yes	Unclear	Yes	Yes	High
Wang et al., 2017^19^	Yes	No	Yes	Yes	Unclear	Yes	Yes	High
Trofa et al., 2019^20^	Yes	Unclear	Yes	Yes	Unclear	Unclear	Unclear	Moderate
Springer et al., 2020^21^	Yes	Yes	Yes	Yes	Yes	Yes	Yes	High

## Discussion

Our analysis shows that online patient education material for the most common sports injuries is at a high reading level. Readability of the included studies was calculated as difficult to read, with no studies reporting a FKGL score under the recommended (no greater than a sixth-grade reading level) threshold for readable patient education material.^35^ This corroborates previous studies that analyzed online patient education material demonstrating poor readability.^19^^,^^27^ Taken together, analysis of the data suggests that many patients may not fully comprehend the continuous stream of online information about a wide range of sports injuries. This may lead to increased hospitalization rate, poor compliance, increased costs, and poor health status.^1^^,^^36^^,^^37^ While decision aids are increasingly being used in orthopaedic practice, aids written beyond the recommended reading level diminishes shared decision-making and the ability of a patient to grasp all attributes of care. Future health-related information should be written by qualified experts, at a level that can be easily understood by patients of all health literacy levels. Surgeons should be more attentive to where patients get their information from and how they interpret it. Accurate, easy-to-understand educational tools can improve efforts to help patients identify misconceptions about treatment options, and to guide patients to choices that are consistent with their values.

The quality and accuracy reported for patient informational resources varied substantially between studies. In general, higher quality sources were more difficult to read (e.g., greater FKGL), which may hinder patients with a poor educational background or English as a second language. Previous studies have found that websites using medical terminology and those that have an advanced reading level are also more accurate.^33^^,^^38^ This confirms a bias that favors patients with greater levels of education and greater health literacy.^11^^,^^12^^,^^38^ While many patients are accessing this information online, it may come up short in its purpose to explain and instruct patients concerning their sports injury and treatment choices. To adequately use the Internet as a resource for health information, clinicians should guide patients to websites that include descriptions of injuries and treatment options that meet their reading level. For example, fifth grade is the average Medicare beneficiary level, and eighth grade is the average U.S. resident reading levels.^39^ Information shared on the internet can impact patients' choices, convictions, and mentalities toward their care. In medicine, qualified experts provide clinical advice; however, most online information is written by people who may not have such qualifications. We found that less than 40% were physician authored. Most patients do not have the right tools to evaluate health literature for biases, unreliability, and inaccurate information; such data can leave patients vulnerable to poor healthcare decisions and misinformation.^40^ Future research may provide updates and more comprehensive insights regarding the characteristics of available patient information. Further, additional work on online patient education of sports injuries should focus on more in-depth assessment of cost utility, impact on total office visit time, and influence on postoperative outcomes, and patient expectations.

### Limitations

There are several limitations to this study. Heterogeneity of the outcome measures and variation in diagnosis and patient characteristics made it difficult to evaluate and compare studies. Furthermore, studies published several years ago or more may be out of date with respect to currently available online patient resources, particularly since the internet is such a massive and constantly changing source of information

## Conclusions

This study demonstrates that online patient information regarding common sports injuries does not match the readability recommendations of the American Medical Association and National Institutes of Health.

## Figures and Tables

**Appendix Table 3:** Embase (Elsevier) Search, September 23, 2020

Search	Query	#
1	'sport injury'/exp OR 'anterior cruciate ligament'/de OR 'anterior cruciate ligament injury'/exp OR 'knee injury'/exp OR 'knee dislocation'/exp OR 'knee meniscus rupture'/exp OR 'knee meniscus'/de OR 'shoulder'/de OR 'shoulder injury'/exp OR 'joint instability'/exp OR 'shoulder dislocation'/exp OR 'rotator cuff'/de OR 'rotator cuff injury'/exp OR 'patella'/de OR 'patella dislocation'/exp OR 'tendinitis'/de OR 'athletic injur∗':ab,ti OR 'sport injur∗':ab,ti OR 'sports injur∗':ab,ti OR 'orthopedic injur∗':ab,ti OR 'orthopaedic injur∗':ab,ti OR 'acl':ab,ti OR 'acl injur∗':ab,ti OR 'acl tear':ab,ti OR 'anterior cruciate ligament':ab,ti OR 'anterior cruciate ligament injur∗':ab,ti OR 'anterior cruciate ligament tear':ab,ti OR 'meniscus':ab,ti OR 'meniscus tear':ab,ti OR 'meniscus injur∗':ab,ti OR 'shoulder':ab,ti OR 'shoulder injur∗':ab,ti OR 'shoulder instability':ab,ti OR 'shoulder dislocation':ab,ti OR 'labral tear':ab,ti OR 'rotator cuff tear':ab,ti OR 'patellar instability':ab,ti OR 'patella':ab,ti OR 'patellar dislocation':ab,ti OR 'tendinitis':ab,ti OR 'tendinopathy':ab,ti OR 'biceps tendinitis':ab,ti OR 'slap tear':ab,ti OR 'slap lesion':ab,ti OR 'knee injur∗':ab,ti OR 'knee dislocation':ab,ti OR 'tibial menisci':ab,ti OR 'tibial meniscus injur∗':ab,ti OR 'shoulder joint':ab,ti OR 'joint instability':ab,ti OR 'rotator cuff':ab,ti OR 'rotator cuff injur∗':ab,ti	225,233
2	'patient education'/exp OR 'educational model'/exp OR 'information dissemination'/de OR 'consumer health information'/exp OR 'health education'/de OR 'publication'/exp OR 'mobile application'/exp OR 'interpersonal communication'/exp OR 'personal digital assistant'/exp OR 'educational technology'/exp OR 'patient education':ab,ti OR 'educational model∗':ab,ti OR 'education model∗':ab,ti OR 'information dissemination':ab,ti OR 'dissemination of information':ab,ti OR 'communication strateg∗':ab,ti OR 'consumer health information':ab,ti OR 'information communication':ab,ti OR 'pamphlet∗':ab,ti OR 'booklet∗':ab,ti OR 'brochure∗':ab,ti OR 'mobile application∗':ab,ti OR 'app':ab,ti OR 'apps':ab,ti OR 'mobile app':ab,ti OR 'mobile apps':ab,ti OR 'smartphone app':ab,ti OR 'smartphone apps':ab,ti OR 'smartphone application∗':ab,ti OR 'communications media':ab,ti OR 'communication':ab,ti OR 'educational technology':ab,ti OR 'education technology':ab,ti OR 'handheld computer∗':ab,ti OR 'mobile phone':ab,ti OR 'smartphone':ab,ti OR 'tablet':ab,ti OR 'patient communication':ab,ti OR 'health communication':ab,ti OR 'health education':ab,ti	1,344,400
3	'patient participation'/exp OR 'shared decision making'/exp OR 'patient care'/exp OR 'rehabilitation'/exp OR 'return to sport'/exp OR 'preoperative period'/exp OR 'postoperative period'/exp OR 'perioperative period'/exp OR 'patient participation':ab,ti OR 'shared decision making':ab,ti OR 'patient-centered':ab,ti OR 'patient centered':ab,ti OR 'patient centered care':ab,ti OR 'decision involvement':ab,ti OR 'patient involvement':ab,ti OR 'patient engagement':ab,ti OR 'rehabilitation':ab,ti OR 'patient expectation':ab,ti OR 'patient expectations':ab,ti OR 'return to sport':ab,ti OR 'return to activity':ab,ti OR (('pre operative':ab,ti OR 'preoperative':ab,ti OR 'postoperative':ab,ti OR 'post operative':ab,ti OR 'peri operative':ab,ti OR 'perioperative':ab,ti OR 'surgery':ab,ti OR 'surgical':ab,ti) AND ('education':ab,ti OR 'communication':ab,ti OR 'rehabilitation':ab,ti OR 'engagement':ab,ti))	2,144,026
4	#1 AND #2 AND #3	2,868

**Appendix Table 1:** PubMed Search, September 23, 2020

Search	Query	#
1	"Athletic Injuries"[Mesh] OR "anterior cruciate ligament"[Mesh] OR "anterior cruciate ligament injuries"[Mesh] OR "knee injuries"[Mesh] OR "Knee Dislocation"[Mesh] OR "tibial meniscus injuries"[Mesh] OR "Meniscus"[Mesh] OR "menisci, tibial"[Mesh] OR "Shoulder"[Mesh] OR "Shoulder Injuries"[Mesh] OR "shoulder joint"[Mesh] OR "joint instability"[Mesh] OR "shoulder dislocation"[Mesh] OR "Rotator Cuff"[Mesh] OR "rotator cuff injuries"[Mesh] OR "Patella"[Mesh] OR "patellar dislocation"[Mesh] OR "Tendinopathy"[Mesh] OR "athletic injur∗"[tiab] OR "sport injur∗"[tiab] OR "sports injur∗"[tiab] OR "orthopedic injur∗"[tiab] OR "orthopaedic injur∗"[tiab] OR "ACL"[tiab] OR "ACL injur∗"[tiab] OR "ACL tear"[tiab] OR "anterior cruciate ligament"[tiab] OR "anterior cruciate ligament injur∗"[tiab] OR "anterior cruciate ligament tear"[tiab] OR "meniscus"[tiab] OR "meniscus tear"[tiab] OR "meniscus injur∗"[tiab] OR “shoulder”[tiab] OR “shoulder injur∗”[tiab] OR "shoulder instability"[tiab] OR "shoulder dislocation"[tiab] OR "labral tear"[tiab] OR "rotator cuff tear"[tiab] OR "patellar instability"[tiab] OR “patella”[tiab] OR “patellar dislocation”[tiab] OR “tendinitis”[tiab] OR “Tendinopathy”[tiab] OR "biceps tendinitis"[tiab] OR "slap tear"[tiab] OR “slap lesion”[tiab] OR "knee injur∗"[tiab] OR "knee dislocation"[tiab] OR "tibial menisci"[tiab] OR "tibial meniscus injur∗"[tiab] OR "shoulder joint"[tiab] OR "joint instability"[tiab] OR “rotator cuff”[tiab] OR "rotator cuff injur∗"[tiab]	192,053
2	"Patient Education as Topic"[Mesh] OR "models, educational"[Mesh] OR "Information Dissemination"[Mesh] OR "Consumer Health Information"[Mesh] OR "health education"[Mesh] OR "Pamphlets"[Mesh] OR "Mobile Applications"[Mesh] OR "Communications Media"[Mesh] OR “Computers, Handheld”[Mesh] OR "Educational Technology"[Mesh] OR "patient education"[tiab] OR "educational model∗"[tiab] OR "education model∗"[tiab] OR "Information Dissemination"[tiab] OR “dissemination of information”[tiab] OR “communication strateg∗”[tiab] OR "Consumer Health Information"[Tiab] OR “information communication”[tiab] OR "Pamphlet∗"[tiab] OR "booklet∗"[tiab] OR “brochure∗”[tiab] OR "Mobile Application∗"[tiab] OR "app∗"[tiab] OR "mobile app∗"[tiab] OR “smartphone app∗”[tiab] OR “smartphone application∗”[tiab] OR "Communications Media"[tiab] OR "communication"[tiab] OR "Educational Technology"[tiab] OR “education technology”[tiab] OR “handheld computer∗”[tiab] OR “mobile phone”[tiab] OR “smartphone”[tiab] OR “tablet”[tiab] OR "patient communication"[tiab] OR "health communication"[Tiab] OR "health education"[tiab]	878,911
3	"patient participation"[Mesh] OR "decision making, shared"[Mesh] OR "patient centered care"[Mesh] OR "rehabilitation"[Mesh] OR "return to sport"[Mesh] OR "preoperative care"[Mesh] OR "preoperative period"[Mesh] OR "perioperative care"[Mesh] OR "postoperative care"[Mesh] OR "patient participation"[Tiab] OR "shared decision making"[Tiab] OR "patient-centered"[Tiab] OR “patient centered”[tiab] OR “patient centered care”[tiab] OR "decision involvement"[Tiab] OR "patient involvement"[Tiab] OR "patient engagement"[Tiab] OR "rehabilitation"[Tiab] OR "patient expectation"[Tiab] OR "patient expectations"[Tiab] OR "return to sport"[Tiab] OR "return to activity"[Tiab] OR (("pre surg∗"[Tiab] OR "pre op∗"[Tiab] OR "presurg∗"[Tiab] OR "before surg∗"[Tiab] OR "preop∗"[Tiab] OR "pre op∗"[Tiab] OR "postop∗"[Tiab] OR "post op∗"[Tiab] OR "post op∗"[Tiab] OR "post surg∗"[Tiab] OR "post surg∗"[Tiab] OR "after surg∗"[Tiab] OR "post procedur∗"[Tiab] OR "peri operative"[Tiab] OR "perioperative"[Tiab]) AND ("education"[Tiab] OR "communication"[Tiab] OR "rehabilitation"[Tiab] OR "engagement"[Tiab]))	648,318
1 AND 2 AND 3	("Athletic Injuries"[MeSH Terms] OR "anterior cruciate ligament"[MeSH Terms] OR "anterior cruciate ligament injuries"[MeSH Terms] OR "knee injuries"[MeSH Terms] OR "Knee Dislocation"[MeSH Terms] OR "tibial meniscus injuries"[MeSH Terms] OR "Meniscus"[MeSH Terms] OR "menisci, tibial"[MeSH Terms] OR "Shoulder"[MeSH Terms] OR "Shoulder Injuries"[MeSH Terms] OR "shoulder joint"[MeSH Terms] OR "joint instability"[MeSH Terms] OR "shoulder dislocation"[MeSH Terms] OR "Rotator Cuff"[MeSH Terms] OR "rotator cuff injuries"[MeSH Terms] OR "Patella"[MeSH Terms] OR "patellar dislocation"[MeSH Terms] OR "Tendinopathy"[MeSH Terms] OR "athletic injur∗"[Title/Abstract] OR "sport injur∗"[Title/Abstract] OR "sports injur∗"[Title/Abstract] OR "orthopedic injur∗"[Title/Abstract] OR "orthopaedic injur∗"[Title/Abstract] OR "ACL"[Title/Abstract] OR "acl injur∗"[Title/Abstract] OR "ACL tear"[Title/Abstract] OR "anterior cruciate ligament"[Title/Abstract] OR "anterior cruciate ligament injur∗"[Title/Abstract] OR "anterior cruciate ligament tear"[Title/Abstract] OR "Meniscus"[Title/Abstract] OR "meniscus tear"[Title/Abstract] OR "meniscus injur∗"[Title/Abstract] OR "Shoulder"[Title/Abstract] OR "shoulder injur∗"[Title/Abstract] OR "shoulder instability"[Title/Abstract] OR "shoulder dislocation"[Title/Abstract] OR "labral tear"[Title/Abstract] OR "rotator cuff tear"[Title/Abstract] OR "patellar instability"[Title/Abstract] OR "Patella"[Title/Abstract] OR "patellar dislocation"[Title/Abstract] OR "tendinitis"[Title/Abstract] OR "Tendinopathy"[Title/Abstract] OR "biceps tendinitis"[Title/Abstract] OR "slap tear"[Title/Abstract] OR "slap lesion"[Title/Abstract] OR "knee injur∗"[Title/Abstract] OR "Knee Dislocation"[Title/Abstract] OR "tibial menisci"[Title/Abstract] OR "tibial meniscus injur∗"[Title/Abstract] OR "shoulder joint"[Title/Abstract] OR "joint instability"[Title/Abstract] OR "Rotator Cuff"[Title/Abstract] OR "rotator cuff injur∗"[Title/Abstract]) AND ("Patient Education as Topic"[MeSH Terms] OR "models, educational"[MeSH Terms] OR "Information Dissemination"[MeSH Terms] OR "Consumer Health Information"[MeSH Terms] OR "health education"[MeSH Terms] OR "Pamphlets"[MeSH Terms] OR "Mobile Applications"[MeSH Terms] OR "Communications Media"[MeSH Terms] OR "computers, handheld"[MeSH Terms] OR "Educational Technology"[MeSH Terms] OR "patient education"[Title/Abstract] OR "educational model∗"[Title/Abstract] OR "education model∗"[Title/Abstract] OR "Information Dissemination"[Title/Abstract] OR "dissemination of information"[Title/Abstract] OR "communication strateg∗"[Title/Abstract] OR "Consumer Health Information"[Title/Abstract] OR "information communication"[Title/Abstract] OR "pamphlet∗"[Title/Abstract] OR "booklet∗"[Title/Abstract] OR "brochure∗"[Title/Abstract] OR "mobile application∗"[Title/Abstract] OR "app"[Title/Abstract] OR "mobile app"[Title/Abstract] OR "mobile apps"[Title/Abstract] OR "smartphone apps"[Title/Abstract] OR "smartphone app"[Title/Abstract] OR "smartphone application∗"[Title/Abstract] OR "Communications Media"[Title/Abstract] OR "communication"[Title/Abstract] OR "Educational Technology"[Title/Abstract] OR "education technology"[Title/Abstract] OR "handheld computer∗"[Title/Abstract] OR "mobile phone"[Title/Abstract] OR "smartphone"[Title/Abstract] OR "tablet"[Title/Abstract] OR "patient communication"[Title/Abstract] OR "health communication"[Title/Abstract] OR "health education"[Title/Abstract]) AND ("patient participation"[MeSH Terms] OR "decision making, shared"[MeSH Terms] OR "patient centered care"[MeSH Terms] OR "rehabilitation"[MeSH Terms] OR "return to sport"[MeSH Terms] OR "preoperative care"[MeSH Terms] OR "preoperative period"[MeSH Terms] OR "perioperative care"[MeSH Terms] OR "postoperative care"[MeSH Terms] OR "patient participation"[Title/Abstract] OR "shared decision making"[Title/Abstract] OR "patient-centered"[Title/Abstract] OR "patient-centered"[Title/Abstract] OR "patient centered care"[Title/Abstract] OR "decision involvement"[Title/Abstract] OR "patient involvement"[Title/Abstract] OR "patient engagement"[Title/Abstract] OR "rehabilitation"[Title/Abstract] OR "patient expectation"[Title/Abstract] OR "patient expectations"[Title/Abstract] OR "return to sport"[Title/Abstract] OR "return to activity"[Title/Abstract] OR (("pre surg∗"[Title/Abstract] OR "pre op∗"[Title/Abstract] OR "presurg∗"[Title/Abstract] OR "before surg∗"[Title/Abstract] OR "preop∗"[Title/Abstract] OR "pre op∗"[Title/Abstract] OR "postop∗"[Title/Abstract] OR "post op∗"[Title/Abstract] OR "post op∗"[Title/Abstract] OR "post surg∗"[Title/Abstract] OR "post surg∗"[Title/Abstract] OR "after surg∗"[Title/Abstract] OR "post procedur∗"[Title/Abstract] OR "peri operative"[Title/Abstract] OR "perioperative"[Title/Abstract]) AND ("education"[Title/Abstract] OR "communication"[Title/Abstract] OR "rehabilitation"[Title/Abstract] OR "engagement"[Title/Abstract])))	722

**Appendix Table 2:** CINAHL Plus with Full Text (EBSCO) Search, September 23, 2020

Search	Query	#
1	TI ( athletic injur∗ OR sport injur∗ OR sports injur∗ OR orthopedic injur∗ OR orthopaedic injur∗ OR ACL OR ACL injur∗ OR ACL tear OR anterior cruciate ligament OR anterior cruciate ligament injur∗ OR anterior cruciate ligament tear OR meniscus OR meniscus tear OR meniscus injur∗ OR shoulder OR shoulder injur∗ OR shoulder instability OR shoulder dislocation OR labral tear OR rotator cuff tear OR patellar instability OR patella OR patellar dislocation OR tendinitis OR Tendinopathy OR biceps tendinitis OR slap tear OR slap lesion OR knee injur∗ OR knee dislocation OR tibial menisci OR tibial meniscus injur∗ OR shoulder joint OR joint instability OR rotator cuff OR rotator cuff injur∗ ) OR AB ( athletic injur∗ OR sport injur∗ OR sports injur∗ OR orthopedic injur∗ OR orthopaedic injur∗ OR ACL OR ACL injur∗ OR ACL tear OR anterior cruciate ligament OR anterior cruciate ligament injur∗ OR anterior cruciate ligament tear OR meniscus OR meniscus tear OR meniscus injur∗ OR shoulder OR shoulder injur∗ OR shoulder instability OR shoulder dislocation OR labral tear OR rotator cuff tear OR patellar instability OR patella OR patellar dislocation OR tendinitis OR Tendinopathy OR biceps tendinitis OR slap tear OR slap lesion OR knee injur∗ OR knee dislocation OR tibial menisci OR tibial meniscus injur∗ OR shoulder joint OR joint instability OR rotator cuff OR rotator cuff injur∗ ) OR MH ( "Anterior Cruciate Ligament" OR "Anterior Cruciate Ligament Injuries" OR "Knee Injuries+" OR "Menisci, Tibial" OR "Meniscal Injuries" OR "Shoulder Instability, Posterior" OR "Shoulder Instability, Multidirectional" OR "Shoulder" OR "Shoulder Dislocation" OR "Shoulder Joint+" OR "Shoulder Injuries+" OR "Joint Instability+" OR "Rotator Cuff Injuries" OR "Patella Dislocation" OR "Athletic Injuries+" )	70,903
2	MH ( "Patient Education+" OR "Models, Educational" OR "Selective Dissemination of Information" OR "Consumer Health Information+" OR "Health Education+" OR "Pamphlets" OR "Mobile Applications" OR "Communications Media+" OR "Computers, Hand-Held+" OR "Educational Technology" ) OR TI ( patient education OR educational model∗ OR education model∗ OR Information Dissemination OR dissemination of information OR communication strateg∗ OR Consumer Health Information OR information communication OR Pamphlet∗ OR booklet∗ OR brochure∗ OR Mobile Application∗ OR app∗ OR mobile app∗ OR smartphone app∗ OR smartphone application∗ OR Communications Media OR communication OR Educational Technology OR education technology OR handheld computer∗ OR mobile phone OR smartphone OR tablet OR patient communication OR health communication OR health education ) OR AB ( patient education OR educational model∗ OR education model∗ OR Information Dissemination OR dissemination of information OR communication strateg∗ OR Consumer Health Information OR information communication OR Pamphlet∗ OR booklet∗ OR brochure∗ OR Mobile Application∗ OR app OR apps OR mobile app OR mobile apps OR smartphone apps OR smartphone app OR smartphone application∗ OR Communications Media OR communication OR Educational Technology OR education technology OR handheld computer∗ OR mobile phone OR smartphone OR tablet OR patient communication OR health communication OR health education )	650,783
3	( TI ( pre operative OR preoperative OR postoperative OR post operative OR peri operative OR perioperative OR surgery OR surgical ) OR AB ( pre operative OR preoperative OR postoperative OR post operative OR peri operative OR perioperative OR surgery OR surgical ) AND TI ( education OR communication OR rehabilitation OR engagement ) OR AB ( education OR communication OR rehabilitation OR engagement ) ) OR ( MH ( "Decision Making, Shared" OR "Patient Centered Care" OR "Rehabilitation+" OR "Sports Re-Entry" OR "Preoperative Education" OR "Preoperative Period+" OR "Preoperative Care+" OR "Perioperative Care+"OR “Postoperative Period” OR "Postoperative Care+" ) OR AB ( patient participation OR shared decision making OR patient-centered OR patient centered OR patient centered care OR decision involvement OR patient involvement OR patient engagement OR rehabilitation OR patient expectation OR patient expectations OR return to sport OR return to activity ) OR TI ( patient participation OR shared decision making OR patient-centered OR patient centered OR patient centered care OR decision involvement OR patient involvement OR patient engagement OR rehabilitation OR patient expectation OR patient expectations OR return to sport OR return to activity ) )	686,596
4	1 AND 2 AND 3	3,652

NOTE. Limiters - abstract available. Search modes - find all my search terms.

## Supplementary Data

ICMJE author disclosure forms
