# Supplementary material for: Online Patient Education Materials for Common Sports Injuries Are Written at Too-High of a Reading Level: A Systematic Review
Source: Arthrosc Sports Med Rehabil. 2022 Feb 11;4(3):e861–75. doi: 10.1016/j.asmr.2021.12.017 (PMC9210373; doi:10.1016/j.asmr.2021.12.017)
Supplement: ICMJE author disclosure forms [file mmc1.pdf]

ICMJE DISCLOSURE FORM

Date: 08/09/2021
Your Name: Mary Mulcahey
Manuscript Title: Patient Education of Common Sports Injuries Between 2000-2020: A Systematic Review
Manuscript number (if known): ____________

In the interest of transparency, we ask you to disclose all relationships/activities/interests listed below that are related to the content of your manuscript. "Related" means any relation with for-profit or not-for-profit third parties whose interests may be affected by the content of the manuscript. Disclosure represents a commitment to transparency and does not necessarily indicate a bias. If you are in doubt about whether to list a relationship/activity/interest, it is preferable that you do so.

The following questions apply to the author's relationships/activities/interests as they relate to the current manuscript only.

The author's relationships/activities/interests should be defined broadly. For example, if your manuscript pertains to the epidemiology of hypertension, you should declare all relationships with manufacturers of antihypertensive medication, even if that medication is not mentioned in the manuscript.

In item #1 below, report all support for the work reported in this manuscript without time limit. For all other items, the time frame for disclosure is the past 36 months.

		**Name all entities with whom you have this relationship or indicate none (add rows as needed)**	**Specifications/Comments (e.g., if payments were made to you or to your institution)**
**Time frame: Since the initial planning of the work**			
1	All support for the present manuscript (e.g., funding, provision of study materials, medical writing, article processing charges, etc.) **No time limit for this item.**	X None	
**Time frame: past 36 months**			
2	Grants or contracts from any entity (if not indicated in item #1 above).	X None	
3	Royalties or licenses	X None	

4	Consulting fees	_X_ None	
5	Payment or honoraria for lectures, presentations, speakers bureaus, manuscript writing or educational events	_X_ None	
6	Payment for expert testimony	_X_ None	
7	Support for attending meetings and/or travel	_X_ None	
8	Patents planned, issued or pending	_X_ None	
9	Participation on a Data Safety Monitoring Board or Advisory Board	_X_ None	
10	Leadership or fiduciary role in other board, society, committee or advocacy group, paid or unpaid	____ None	AAOS: Board or committee member<br>American Journal of Sports Medicine Electronic Media: Editorial or governing board<br>American Orthopaedic Association: Board or committee member<br>American Orthopaedic Society for Sports Medicine: Board or committee member<br>Arthroscopy: Editorial or governing board<br>Arthroscopy Association of North America: Board or committee member<br>Ortho Info: Editorial or governing board<br>Ruth Jackson Orthopaedic Society: Board or committee member<br>The Forum: Board or committee member
11	Stock or stock options	_X_ None	
12	Receipt of equipment, materials, drugs, medical writing, gifts or other services	_X_ None	
13	Other financial or non-financial interests	____ None	Arthrex, Inc: Paid presenter or speaker

Please place an "X" next to the following statement to indicate your agreement:

_X_ **I certify that I have answered every question and have not altered the wording of any of the questions on this form.**

ICMJE DISCLOSURE FORM

Date: 08/09/2021

Your Name: Samantha O'Connell

Manuscript Title: Patient Education of Common Sports Injuries Between 2000-2020: A Systematic Review

Manuscript number (if known):________________

In the interest of transparency, we ask you to disclose all relationships/activities/interests listed below that are related to the content of your manuscript. "Related" means any relation with for-profit or not-for-profit third parties whose interests may be affected by the content of the manuscript. Disclosure represents a commitment to transparency and does not necessarily indicate a bias. If you are in doubt about whether to list a relationship/activity/interest, it is preferable that you do so.

The following questions apply to the author's relationships/activities/interests as they relate to the <u>current manuscript only</u>.

The author's relationships/activities/interests should be <u>defined broadly</u>. For example, if your manuscript pertains to the epidemiology of hypertension, you should declare all relationships with manufacturers of antihypertensive medication, even if that medication is not mentioned in the manuscript.

In item #1 below, report all support for the work reported in this manuscript without time limit. For all other items, the time frame for disclosure is the past 36 months.

		**Name all entities with whom you have this relationship or indicate none (add rows as needed)**	**Specifications/Comments (e.g., if payments were made to you or to your institution)**
**Time frame: Since the initial planning of the work**			
1	All support for the present manuscript (e.g., funding, provision of study materials, medical writing, article processing charges, etc.) **No time limit for this item.**	X None	
**Time frame: past 36 months**			
2	Grants or contracts from any entity (if not indicated in item #1 above).	X None	
3	Royalties or licenses	X None	

4	Consulting fees	_X_ None	
5	Payment or honoraria for lectures, presentations, speakers bureaus, manuscript writing or educational events	_X_ None	
6	Payment for expert testimony	_X_ None	
7	Support for attending meetings and/or travel	_X_ None	
8	Patents planned, issued or pending	_X_ None	
9	Participation on a Data Safety Monitoring Board or Advisory Board	_X_ None	
10	Leadership or fiduciary role in other board, society, committee or advocacy group, paid or unpaid	_X_ None	
11	Stock or stock options	_X_ None	
12	Receipt of equipment, materials, drugs, medical writing, gifts or other services	_X_ None	
13	Other financial or non-financial interests	_X_ None	

Please place an "X" next to the following statement to indicate your agreement:

_X_ **I certify that I have answered every question and have not altered the wording of any of the questions on this form.**

ICMJE DISCLOSURE FORM

Date: 08/09/2021
Your Name: Aaron Alokozai
Manuscript Title: Patient Education of Common Sports Injuries Between 2000-2020: A Systematic Review
Manuscript number (if known): ______

In the interest of transparency, we ask you to disclose all relationships/activities/interests listed below that are related to the content of your manuscript. "Related" means any relation with for-profit or not-for-profit third parties whose interests may be affected by the content of the manuscript. Disclosure represents a commitment to transparency and does not necessarily indicate a bias. If you are in doubt about whether to list a relationship/activity/interest, it is preferable that you do so.

The following questions apply to the author's relationships/activities/interests as they relate to the current manuscript only.

The author's relationships/activities/interests should be defined broadly. For example, if your manuscript pertains to the epidemiology of hypertension, you should declare all relationships with manufacturers of antihypertensive medication, even if that medication is not mentioned in the manuscript.

In item #1 below, report all support for the work reported in this manuscript without time limit. For all other items, the time frame for disclosure is the past 36 months.

		**Name all entities with whom you have this relationship or indicate none (add rows as needed)**	**Specifications/Comments (e.g., if payments were made to you or to your institution)**
**Time frame: Since the initial planning of the work**			
1	All support for the present manuscript (e.g., funding, provision of study materials, medical writing, article processing charges, etc.)<br>**No time limit for this item.**	X None	
**Time frame: past 36 months**			
2	Grants or contracts from any entity (if not indicated in item #1 above).	X None	
3	Royalties or licenses	X None	

4	Consulting fees	__X__ None	
5	Payment or honoraria for lectures, presentations, speakers bureaus, manuscript writing or educational events	__X__ None	
6	Payment for expert testimony	__X__ None	
7	Support for attending meetings and/or travel	__X__ None	
8	Patents planned, issued or pending	__X__ None	
9	Participation on a Data Safety Monitoring Board or Advisory Board	__X__ None	
10	Leadership or fiduciary role in other board, society, committee or advocacy group, paid or unpaid	__X__ None	
11	Stock or stock options	__X__ None	
12	Receipt of equipment, materials, drugs, medical writing, gifts or other services	__X__ None	
13	Other financial or non-financial interests	__X__ None	

Please place an "X" next to the following statement to indicate your agreement:

__X__ **I certify that I have answered every question and have not altered the wording of any of the questions on this form.**

ICMJE DISCLOSURE FORM

Date: 08/09/2021
Your Name: Youssef Abdullah
Manuscript Title: Patient Education of Common Sports Injuries Between 2000-2020: A Systematic Review
Manuscript number (if known):______

In the interest of transparency, we ask you to disclose all relationships/activities/interests listed below that are related to the content of your manuscript. "Related" means any relation with for-profit or not-for-profit third parties whose interests may be affected by the content of the manuscript. Disclosure represents a commitment to transparency and does not necessarily indicate a bias. If you are in doubt about whether to list a relationship/activity/interest, it is preferable that you do so.

The following questions apply to the author's relationships/activities/interests as they relate to the current manuscript only.

The author's relationships/activities/interests should be defined broadly. For example, if your manuscript pertains to the epidemiology of hypertension, you should declare all relationships with manufacturers of antihypertensive medication, even if that medication is not mentioned in the manuscript.

In item #1 below, report all support for the work reported in this manuscript without time limit. For all other items, the time frame for disclosure is the past 36 months.

		**Name all entities with whom you have this relationship or indicate none (add rows as needed)**	**Specifications/Comments (e.g., if payments were made to you or to your institution)**
		**Time frame: Since the initial planning of the work**	
1	All support for the present manuscript (e.g., funding, provision of study materials, medical writing, article processing charges, etc.) **No time limit for this item.**	x None	
		**Time frame: past 36 months**	
2	Grants or contracts from any entity (if not indicated in item #1 above).	x None	
3	Royalties or licenses	x None	

4	Consulting fees	_X_ None	
5	Payment or honoraria for lectures, presentations, speakers bureaus, manuscript writing or educational events	_X_ None	
6	Payment for expert testimony	_X_ None	
7	Support for attending meetings and/or travel	_X_ None	
8	Patents planned, issued or pending	_X_ None	
9	Participation on a Data Safety Monitoring Board or Advisory Board	_X_ None	
10	Leadership or fiduciary role in other board, society, committee or advocacy group, paid or unpaid	_X_ None	
11	Stock or stock options	_X_ None	
12	Receipt of equipment, materials, drugs, medical writing, gifts or other services	_X_ None	
13	Other financial or non-financial interests	_X_ None	

Please place an "X" next to the following statement to indicate your agreement:

_X_ **I certify that I have answered every question and have not altered the wording of any of the questions on this form.**
