# Supplementary material for: Online Patient Education Materials for Common Sports Injuries Are Written at Too-High of a Reading Level: A Systematic Review
Source: Arthrosc Sports Med Rehabil. 2022 Feb 11;4(3):e861–75. doi: 10.1016/j.asmr.2021.12.017 (PMC9210373; doi:10.1016/j.asmr.2021.12.017)
Supplement: Supplementary Material [file mmc2.docx]

PubMed 09/23/2020 Search

| Search | Query | # |
| --- | --- | --- |
| 1 | "Athletic Injuries"[Mesh] OR "anterior cruciate ligament"[Mesh] OR "anterior cruciate ligament injuries"[Mesh] OR "knee injuries"[Mesh] OR "Knee Dislocation"[Mesh] OR "tibial meniscus injuries"[Mesh] OR "Meniscus"[Mesh] OR "menisci, tibial"[Mesh] OR "Shoulder"[Mesh] OR "Shoulder Injuries"[Mesh] OR "shoulder joint"[Mesh] OR "joint instability"[Mesh] OR "shoulder dislocation"[Mesh] OR "Rotator Cuff"[Mesh] OR "rotator cuff injuries"[Mesh] OR "Patella"[Mesh] OR "patellar dislocation"[Mesh] OR "Tendinopathy"[Mesh] OR "athletic injur*"[tiab] OR "sport injur*"[tiab] OR "sports injur*"[tiab] OR "orthopedic injur*"[tiab] OR "orthopaedic injur*"[tiab] OR "ACL"[tiab] OR "ACL injur*"[tiab] OR "ACL tear"[tiab] OR "anterior cruciate ligament"[tiab] OR "anterior cruciate ligament injur*"[tiab] OR "anterior cruciate ligament tear"[tiab] OR "meniscus"[tiab] OR "meniscus tear"[tiab] OR "meniscus injur*"[tiab] OR “shoulder”[tiab] OR “shoulder injur*”[tiab] OR "shoulder instability"[tiab] OR "shoulder dislocation"[tiab] OR "labral tear"[tiab] OR "rotator cuff tear"[tiab] OR "patellar instability"[tiab] OR “patella”[tiab] OR “patellar dislocation”[tiab] OR “tendinitis”[tiab] OR “Tendinopathy”[tiab] OR "biceps tendinitis"[tiab] OR "slap tear"[tiab] OR “slap lesion”[tiab] OR "knee injur*"[tiab] OR "knee dislocation"[tiab] OR "tibial menisci"[tiab] OR "tibial meniscus injur*"[tiab] OR "shoulder joint"[tiab] OR "joint instability"[tiab] OR “rotator cuff”[tiab] OR "rotator cuff injur*"[tiab] | 192,053 |
| 2 | "Patient Education as Topic"[Mesh] OR "models, educational"[Mesh] OR "Information Dissemination"[Mesh] OR "Consumer Health Information"[Mesh] OR "health education"[Mesh] OR "Pamphlets"[Mesh] OR "Mobile Applications"[Mesh] OR "Communications Media"[Mesh] OR “Computers, Handheld”[Mesh] OR "Educational Technology"[Mesh] OR "patient education"[tiab] OR "educational model*"[tiab] OR "education model*"[tiab] OR "Information Dissemination"[tiab] OR “dissemination of information”[tiab] OR “communication strateg*”[tiab] OR "Consumer Health Information"[Tiab] OR “information communication”[tiab] OR "Pamphlet*"[tiab] OR "booklet*"[tiab] OR “brochure*”[tiab] OR "Mobile Application*"[tiab] OR "app*"[tiab] OR "mobile app*"[tiab] OR “smartphone app*”[tiab] OR “smartphone application*”[tiab] OR "Communications Media"[tiab] OR "communication"[tiab] OR "Educational Technology"[tiab] OR “education technology”[tiab] OR “handheld computer*”[tiab] OR “mobile phone”[tiab] OR “smartphone”[tiab] OR “tablet”[tiab] OR "patient communication"[tiab] OR "health communication"[Tiab] OR "health education"[tiab] | 878,911 |
| 3 | "patient participation"[Mesh] OR "decision making, shared"[Mesh] OR "patient centered care"[Mesh] OR "rehabilitation"[Mesh] OR "return to sport"[Mesh] OR "preoperative care"[Mesh] OR "preoperative period"[Mesh] OR "perioperative care"[Mesh] OR "postoperative care"[Mesh] OR "patient participation"[Tiab] OR "shared decision making"[Tiab] OR "patient-centered"[Tiab] OR “patient centered”[tiab] OR “patient centered care”[tiab] OR "decision involvement"[Tiab] OR "patient involvement"[Tiab] OR "patient engagement"[Tiab] OR "rehabilitation"[Tiab] OR "patient expectation"[Tiab] OR "patient expectations"[Tiab] OR "return to sport"[Tiab] OR "return to activity"[Tiab] OR (("pre surg*"[Tiab] OR "pre op*"[Tiab] OR "presurg*"[Tiab] OR "before surg*"[Tiab] OR "preop*"[Tiab] OR "pre op*"[Tiab] OR "postop*"[Tiab] OR "post op*"[Tiab] OR "post op*"[Tiab] OR "post surg*"[Tiab] OR "post surg*"[Tiab] OR "after surg*"[Tiab] OR "post procedur*"[Tiab] OR "peri operative"[Tiab] OR "perioperative"[Tiab]) AND ("education"[Tiab] OR "communication"[Tiab] OR "rehabilitation"[Tiab] OR "engagement"[Tiab])) | 648,318 |
| 1  AND  2  AND  3 | ("Athletic Injuries"[MeSH Terms] OR "anterior cruciate ligament"[MeSH Terms] OR "anterior cruciate ligament injuries"[MeSH Terms] OR "knee injuries"[MeSH Terms] OR "Knee Dislocation"[MeSH Terms] OR "tibial meniscus injuries"[MeSH Terms] OR "Meniscus"[MeSH Terms] OR "menisci, tibial"[MeSH Terms] OR "Shoulder"[MeSH Terms] OR "Shoulder Injuries"[MeSH Terms] OR "shoulder joint"[MeSH Terms] OR "joint instability"[MeSH Terms] OR "shoulder dislocation"[MeSH Terms] OR "Rotator Cuff"[MeSH Terms] OR "rotator cuff injuries"[MeSH Terms] OR "Patella"[MeSH Terms] OR "patellar dislocation"[MeSH Terms] OR "Tendinopathy"[MeSH Terms] OR "athletic injur*"[Title/Abstract] OR "sport injur*"[Title/Abstract] OR "sports injur*"[Title/Abstract] OR "orthopedic injur*"[Title/Abstract] OR "orthopaedic injur*"[Title/Abstract] OR "ACL"[Title/Abstract] OR "acl injur*"[Title/Abstract] OR "ACL tear"[Title/Abstract] OR "anterior cruciate ligament"[Title/Abstract] OR "anterior cruciate ligament injur*"[Title/Abstract] OR "anterior cruciate ligament tear"[Title/Abstract] OR "Meniscus"[Title/Abstract] OR "meniscus tear"[Title/Abstract] OR "meniscus injur*"[Title/Abstract] OR "Shoulder"[Title/Abstract] OR "shoulder injur*"[Title/Abstract] OR "shoulder instability"[Title/Abstract] OR "shoulder dislocation"[Title/Abstract] OR "labral tear"[Title/Abstract] OR "rotator cuff tear"[Title/Abstract] OR "patellar instability"[Title/Abstract] OR "Patella"[Title/Abstract] OR "patellar dislocation"[Title/Abstract] OR "tendinitis"[Title/Abstract] OR "Tendinopathy"[Title/Abstract] OR "biceps tendinitis"[Title/Abstract] OR "slap tear"[Title/Abstract] OR "slap lesion"[Title/Abstract] OR "knee injur*"[Title/Abstract] OR "Knee Dislocation"[Title/Abstract] OR "tibial menisci"[Title/Abstract] OR "tibial meniscus injur*"[Title/Abstract] OR "shoulder joint"[Title/Abstract] OR "joint instability"[Title/Abstract] OR "Rotator Cuff"[Title/Abstract] OR "rotator cuff injur*"[Title/Abstract]) AND ("Patient Education as Topic"[MeSH Terms] OR "models, educational"[MeSH Terms] OR "Information Dissemination"[MeSH Terms] OR "Consumer Health Information"[MeSH Terms] OR "health education"[MeSH Terms] OR "Pamphlets"[MeSH Terms] OR "Mobile Applications"[MeSH Terms] OR "Communications Media"[MeSH Terms] OR "computers, handheld"[MeSH Terms] OR "Educational Technology"[MeSH Terms] OR "patient education"[Title/Abstract] OR "educational model*"[Title/Abstract] OR "education model*"[Title/Abstract] OR "Information Dissemination"[Title/Abstract] OR "dissemination of information"[Title/Abstract] OR "communication strateg*"[Title/Abstract] OR "Consumer Health Information"[Title/Abstract] OR "information communication"[Title/Abstract] OR "pamphlet*"[Title/Abstract] OR "booklet*"[Title/Abstract] OR "brochure*"[Title/Abstract] OR "mobile application*"[Title/Abstract] OR "app"[Title/Abstract] OR "mobile app"[Title/Abstract] OR "mobile apps"[Title/Abstract] OR "smartphone apps"[Title/Abstract] OR "smartphone app"[Title/Abstract] OR "smartphone application*"[Title/Abstract] OR "Communications Media"[Title/Abstract] OR "communication"[Title/Abstract] OR "Educational Technology"[Title/Abstract] OR "education technology"[Title/Abstract] OR "handheld computer*"[Title/Abstract] OR "mobile phone"[Title/Abstract] OR "smartphone"[Title/Abstract] OR "tablet"[Title/Abstract] OR "patient communication"[Title/Abstract] OR "health communication"[Title/Abstract] OR "health education"[Title/Abstract]) AND ("patient participation"[MeSH Terms] OR "decision making, shared"[MeSH Terms] OR "patient centered care"[MeSH Terms] OR "rehabilitation"[MeSH Terms] OR "return to sport"[MeSH Terms] OR "preoperative care"[MeSH Terms] OR "preoperative period"[MeSH Terms] OR "perioperative care"[MeSH Terms] OR "postoperative care"[MeSH Terms] OR "patient participation"[Title/Abstract] OR "shared decision making"[Title/Abstract] OR "patient-centered"[Title/Abstract] OR "patient-centered"[Title/Abstract] OR "patient centered care"[Title/Abstract] OR "decision involvement"[Title/Abstract] OR "patient involvement"[Title/Abstract] OR "patient engagement"[Title/Abstract] OR "rehabilitation"[Title/Abstract] OR "patient expectation"[Title/Abstract] OR "patient expectations"[Title/Abstract] OR "return to sport"[Title/Abstract] OR "return to activity"[Title/Abstract] OR (("pre surg*"[Title/Abstract] OR "pre op*"[Title/Abstract] OR "presurg*"[Title/Abstract] OR "before surg*"[Title/Abstract] OR "preop*"[Title/Abstract] OR "pre op*"[Title/Abstract] OR "postop*"[Title/Abstract] OR "post op*"[Title/Abstract] OR "post op*"[Title/Abstract] OR "post surg*"[Title/Abstract] OR "post surg*"[Title/Abstract] OR "after surg*"[Title/Abstract] OR "post procedur*"[Title/Abstract] OR "peri operative"[Title/Abstract] OR "perioperative"[Title/Abstract]) AND ("education"[Title/Abstract] OR "communication"[Title/Abstract] OR "rehabilitation"[Title/Abstract] OR "engagement"[Title/Abstract]))) | 722 |

CINAHL Plus with Full Text (EBSCO) 09/23/2020 Search
Limiters - Abstract Available
Search modes - Find all my search terms

| Search | Query | # |
| --- | --- | --- |
| 1 | TI ( athletic injur* OR sport injur* OR sports injur* OR orthopedic injur* OR orthopaedic injur* OR ACL OR ACL injur* OR ACL tear OR anterior cruciate ligament OR anterior cruciate ligament injur* OR anterior cruciate ligament tear OR meniscus OR meniscus tear OR meniscus injur* OR shoulder OR shoulder injur* OR shoulder instability OR shoulder dislocation OR labral tear OR rotator cuff tear OR patellar instability OR patella OR patellar dislocation OR tendinitis OR Tendinopathy OR biceps tendinitis OR slap tear OR slap lesion OR knee injur* OR knee dislocation OR tibial menisci OR tibial meniscus injur* OR shoulder joint OR joint instability OR rotator cuff OR rotator cuff injur* ) OR AB ( athletic injur* OR sport injur* OR sports injur* OR orthopedic injur* OR orthopaedic injur* OR ACL OR ACL injur* OR ACL tear OR anterior cruciate ligament OR anterior cruciate ligament injur* OR anterior cruciate ligament tear OR meniscus OR meniscus tear OR meniscus injur* OR shoulder OR shoulder injur* OR shoulder instability OR shoulder dislocation OR labral tear OR rotator cuff tear OR patellar instability OR patella OR patellar dislocation OR tendinitis OR Tendinopathy OR biceps tendinitis OR slap tear OR slap lesion OR knee injur* OR knee dislocation OR tibial menisci OR tibial meniscus injur* OR shoulder joint OR joint instability OR rotator cuff OR rotator cuff injur* ) OR MH ( "Anterior Cruciate Ligament" OR "Anterior Cruciate Ligament Injuries" OR "Knee Injuries+" OR "Menisci, Tibial" OR "Meniscal Injuries" OR "Shoulder Instability, Posterior" OR "Shoulder Instability, Multidirectional" OR "Shoulder" OR "Shoulder Dislocation" OR "Shoulder Joint+" OR "Shoulder Injuries+" OR "Joint Instability+" OR "Rotator Cuff Injuries" OR "Patella Dislocation" OR "Athletic Injuries+" ) | 70,903 |
| 2 | MH ( "Patient Education+" OR "Models, Educational" OR "Selective Dissemination of Information" OR "Consumer Health Information+" OR "Health Education+" OR "Pamphlets" OR "Mobile Applications" OR "Communications Media+" OR "Computers, Hand-Held+" OR "Educational Technology" ) OR TI ( patient education OR educational model* OR education model* OR Information Dissemination OR dissemination of information OR communication strateg* OR Consumer Health Information OR information communication OR Pamphlet* OR booklet* OR brochure* OR Mobile Application* OR app* OR mobile app* OR smartphone app* OR smartphone application* OR Communications Media OR communication OR Educational Technology OR education technology OR handheld computer* OR mobile phone OR smartphone OR tablet OR patient communication OR health communication OR health education ) OR AB ( patient education OR educational model* OR education model* OR Information Dissemination OR dissemination of information OR communication strateg* OR Consumer Health Information OR information communication OR Pamphlet* OR booklet* OR brochure* OR Mobile Application* OR app OR apps OR mobile app OR mobile apps OR smartphone apps OR smartphone app OR smartphone application* OR Communications Media OR communication OR Educational Technology OR education technology OR handheld computer* OR mobile phone OR smartphone OR tablet OR patient communication OR health communication OR health education ) | 650,783 |
| 3 | ( TI ( pre operative OR preoperative OR postoperative OR post operative OR peri operative OR perioperative OR surgery OR surgical ) OR AB ( pre operative OR preoperative OR postoperative OR post operative OR peri operative OR perioperative OR surgery OR surgical ) AND TI ( education OR communication OR rehabilitation OR engagement ) OR AB ( education OR communication OR rehabilitation OR engagement ) ) OR ( MH ( "Decision Making, Shared" OR "Patient Centered Care" OR "Rehabilitation+" OR "Sports Re-Entry" OR "Preoperative Education" OR "Preoperative Period+" OR "Preoperative Care+" OR "Perioperative Care+"OR “Postoperative Period” OR "Postoperative Care+" ) OR AB ( patient participation OR shared decision making OR patient-centered OR patient centered OR patient centered care OR decision involvement OR patient involvement OR patient engagement OR rehabilitation OR patient expectation OR patient expectations OR return to sport OR return to activity ) OR TI ( patient participation OR shared decision making OR patient-centered OR patient centered OR patient centered care OR decision involvement OR patient involvement OR patient engagement OR rehabilitation OR patient expectation OR patient expectations OR return to sport OR return to activity ) ) | 686,596 |
| 4 | 1 AND 2 AND 3 | 3,652 |

Embase (Elsevier) Search – 09/23/2020

| Search | Query | # |
| --- | --- | --- |
| 1 | 'sport injury'/exp OR 'anterior cruciate ligament'/de OR 'anterior cruciate ligament injury'/exp OR 'knee injury'/exp OR 'knee dislocation'/exp OR 'knee meniscus rupture'/exp OR 'knee meniscus'/de OR 'shoulder'/de OR 'shoulder injury'/exp OR 'joint instability'/exp OR 'shoulder dislocation'/exp OR 'rotator cuff'/de OR 'rotator cuff injury'/exp OR 'patella'/de OR 'patella dislocation'/exp OR 'tendinitis'/de OR 'athletic injur*':ab,ti OR 'sport injur*':ab,ti OR 'sports injur*':ab,ti OR 'orthopedic injur*':ab,ti OR 'orthopaedic injur*':ab,ti OR 'acl':ab,ti OR 'acl injur*':ab,ti OR 'acl tear':ab,ti OR 'anterior cruciate ligament':ab,ti OR 'anterior cruciate ligament injur*':ab,ti OR 'anterior cruciate ligament tear':ab,ti OR 'meniscus':ab,ti OR 'meniscus tear':ab,ti OR 'meniscus injur*':ab,ti OR 'shoulder':ab,ti OR 'shoulder injur*':ab,ti OR 'shoulder instability':ab,ti OR 'shoulder dislocation':ab,ti OR 'labral tear':ab,ti OR 'rotator cuff tear':ab,ti OR 'patellar instability':ab,ti OR 'patella':ab,ti OR 'patellar dislocation':ab,ti OR 'tendinitis':ab,ti OR 'tendinopathy':ab,ti OR 'biceps tendinitis':ab,ti OR 'slap tear':ab,ti OR 'slap lesion':ab,ti OR 'knee injur*':ab,ti OR 'knee dislocation':ab,ti OR 'tibial menisci':ab,ti OR 'tibial meniscus injur*':ab,ti OR 'shoulder joint':ab,ti OR 'joint instability':ab,ti OR 'rotator cuff':ab,ti OR 'rotator cuff injur*':ab,ti | 225,233 |
| 2 | 'patient education'/exp OR 'educational model'/exp OR 'information dissemination'/de OR 'consumer health information'/exp OR 'health education'/de OR 'publication'/exp OR 'mobile application'/exp OR 'interpersonal communication'/exp OR 'personal digital assistant'/exp OR 'educational technology'/exp OR 'patient education':ab,ti OR 'educational model*':ab,ti OR 'education model*':ab,ti OR 'information dissemination':ab,ti OR 'dissemination of information':ab,ti OR 'communication strateg*':ab,ti OR 'consumer health information':ab,ti OR 'information communication':ab,ti OR 'pamphlet*':ab,ti OR 'booklet*':ab,ti OR 'brochure*':ab,ti OR 'mobile application*':ab,ti OR 'app':ab,ti OR 'apps':ab,ti OR 'mobile app':ab,ti OR 'mobile apps':ab,ti OR 'smartphone app':ab,ti OR 'smartphone apps':ab,ti OR 'smartphone application*':ab,ti OR 'communications media':ab,ti OR 'communication':ab,ti OR 'educational technology':ab,ti OR 'education technology':ab,ti OR 'handheld computer*':ab,ti OR 'mobile phone':ab,ti OR 'smartphone':ab,ti OR 'tablet':ab,ti OR 'patient communication':ab,ti OR 'health communication':ab,ti OR 'health education':ab,ti | 1,344,400 |
| 3 | 'patient participation'/exp OR 'shared decision making'/exp OR 'patient care'/exp OR 'rehabilitation'/exp OR 'return to sport'/exp OR 'preoperative period'/exp OR 'postoperative period'/exp OR 'perioperative period'/exp OR 'patient participation':ab,ti OR 'shared decision making':ab,ti OR 'patient-centered':ab,ti OR 'patient centered':ab,ti OR 'patient centered care':ab,ti OR 'decision involvement':ab,ti OR 'patient involvement':ab,ti OR 'patient engagement':ab,ti OR 'rehabilitation':ab,ti OR 'patient expectation':ab,ti OR 'patient expectations':ab,ti OR 'return to sport':ab,ti OR 'return to activity':ab,ti OR (('pre operative':ab,ti OR 'preoperative':ab,ti OR 'postoperative':ab,ti OR 'post operative':ab,ti OR 'peri operative':ab,ti OR 'perioperative':ab,ti OR 'surgery':ab,ti OR 'surgical':ab,ti) AND ('education':ab,ti OR 'communication':ab,ti OR 'rehabilitation':ab,ti OR 'engagement':ab,ti)) | 2,144,026 |
| 4 | #1 AND #2 AND #3 | **2,868** |
